# Efficacy and safety of twice-daily rabeprazole maintenance therapy for patients with reflux esophagitis refractory to standard once-daily proton pump inhibitor: the Japan-based EXTEND study

**DOI:** 10.1007/s00535-017-1417-z

**Published:** 2017-11-29

**Authors:** Yoshikazu Kinoshita, Mototsugu Kato, Mitsuhiro Fujishiro, Hironori Masuyama, Ryo Nakata, Hisanori Abe, Shinji Kumagai, Yasushi Fukushima, Yoshiumi Okubo, Seiichiro Hojo, Motoyasu Kusano

**Affiliations:** 1 Department of Internal Medicine II, Faculty of Medicine, Shimane University, 89-1, Enya-cho, Izumo, Shimane 693-8501 Japan; 2 Department of Gastroenterology, National Hospital Organization Hakodate Hospital, Hakodate, Hokkaido Japan; 3 Department of Endoscopy and Endoscopic Surgery, Graduate School of Medicine, The University of Tokyo, Tokyo, Japan; 4 Masuyama Gastrointestinal Clinic, Ohtawara, Tochigi Japan; 5 Japanese Red Cross Medical Center, Tokyo, Japan; 6 Arita GI Hospital, Oita, Oita Japan; 7 Nakajima Hospital, Sendai, Miyagi Japan; 8 Tokyo-Eki Center-Building Clinic, Tokyo, Japan; 9 Clinical Development Department, EA Pharma Co., Ltd, Tokyo, Japan; 10 Clinical Data Science Department, Eisai Co., Ltd, Tokyo, Japan; 11 Department of Gastroenterology, Gunma University Hospital, Maebashi, Gunma Japan

**Keywords:** Endoscopy, GERD, Los Angeles Classification, Proton pump inhibitor, Reflux esophagitis

## Abstract

**Background:**

Rabeprazole at 10 or 20 mg twice daily (b.i.d.) has been reported to be highly effective in the treatment of proton pump inhibitor (PPI)-resistant reflux esophagitis (RE) that is refractory to the standard once-daily PPI regimen. We evaluated the efficacy and safety of rabeprazole maintenance therapy at 10 mg once daily (q.d.) or b.i.d. for longer than 8 weeks.

**Methods:**

Patients with RE refractory to standard PPI regimens for at least 8 weeks were enrolled. They were treated with rabeprazole at 10 or 20 mg b.i.d. for 8 weeks during the open-label treatment period. After endoscopic examination, those with confirmed healing entered the subsequent double-blind maintenance therapy. During this period, the subjects were randomized to receive rabeprazole 10 mg q.d. (control) or 10 mg b.i.d. The primary endpoint was the endoscopic no-recurrence rate at Week 52.

**Results:**

In total, 517 subjects entered the treatment, and 359 subjects continued on maintenance therapy. The full analysis set for central assessment included 343 subjects. The no-recurrence rate at Week 52 was significantly higher in the b.i.d. group (73.9%; *p* < 0.001, χ^2^ test) than in the q.d. group (44.8%). In particular, the b.i.d. regimen was more effective in all subgroups with Los Angeles Classification Grade B to D at treatment entry.

**Conclusions:**

In the maintenance treatment of PPI-resistant RE, rabeprazole at 10 mg b.i.d. exerted a stronger recurrence-preventing effect than 10 mg q.d. over 52 weeks. No particular safety issues were noted during long-term administration.

*ClinicalTrials.gov number*: NCT02135107.

## Introduction

The numbers of patients suffering from reflux esophagitis (RE) have reportedly been increasing since the 1990s in Japan. The causes include a higher prevalence of risk factors for RE due to rising obesity rates [1], elevated gastric acid secretion associated with dietary and other lifestyle changes, and increased gastric acid secretion due to a lower prevalence of *Helicobacter pylori*-infected patients [2–4]. Proton pump inhibitors (PPI) have been proven to be highly effective in the treatment of RE, but the disease eventually recurs in 70–80% of patients [5]. Thus, maintenance therapy is important for prevention of recurrence and complications [6]. Maintenance therapy with PPI is regarded as highly effective [7] and as providing favorable cost–benefit performance. Specifically, rabeprazole taken once daily (q.d). has been shown to be effective as maintenance therapy for up to 5 years [8].

While PPI is widely used as the first-line therapy for RE, 6–15% of patients have PPI-refractory RE, such that a standard once-daily PPI regimen is ineffective for healing esophageal mucosal breaks or improving symptoms [9]. In these patients, lower quality of life (QOL) and work loss are reported [10, 11]. PPI-refractory RE might be attributable to nocturnal gastric acid breakthrough and the extensive metabolizer (EM) phenotype associated with the cytochrome P450 2C19 (CYP2C19) genotype. Standard PPI regimens are reportedly insufficient for suppressing nocturnal acid reflux in particular [12, 13]. Patients with PPI-refractory RE are considered to have more frequent nocturnal reflux symptoms and sleep disorders due to longer retention of regurgitated gastric acid in the esophagus [14, 15].

Rabeprazole is reportedly more effective in suppressing nocturnal gastric acid secretion as a twice-daily (b.i.d.) regimen than as a q.d. regimen at the same daily dose [16]. In addition, the efficacies of PPI differ among individuals depending on genetic polymorphism in the hepatic drug metabolizing enzyme CYP2C19, although the impact of rabeprazole is considered to be less significant than that of other PPIs [17].

Based on reports that RE patients in the remission phase have elevated basal secretion similar to that in the active phase [18, 19], maintenance therapy for PPI-refractory RE requires continued potent inhibition of acid secretion after initial treatment.

The Japanese Society of Gastroenterology Clinical Practice Guidelines for Gastroesophageal Reflux Disease (GERD) recommend the rabeprazole b.i.d. regimen for treating PPI-refractory RE [16, 20], but evidence supporting efficacy is available only up to 8 weeks. To demonstrate the efficacy of the divided dosing regimen for longer than 8 weeks, this study was conducted to verify the efficacy and safety of rabeprazole 10 mg b.i.d. as maintenance therapy for PPI-refractory RE.

## Methods

### Study design and protocol

This was a multicenter, randomized, parallel-group, double-blind comparative study evaluating the efficacy and safety of rabeprazole 10 mg b.i.d. for 52 weeks as maintenance therapy. In total, 85 Japanese sites participated in this study.

This study was conducted in compliance with the ethical principles based on the Declaration of Helsinki—Ethical Principles, the Pharmaceuticals Affairs Law, and the standard operating procedures and study protocol established by the sponsor.

**Fig. 1:**
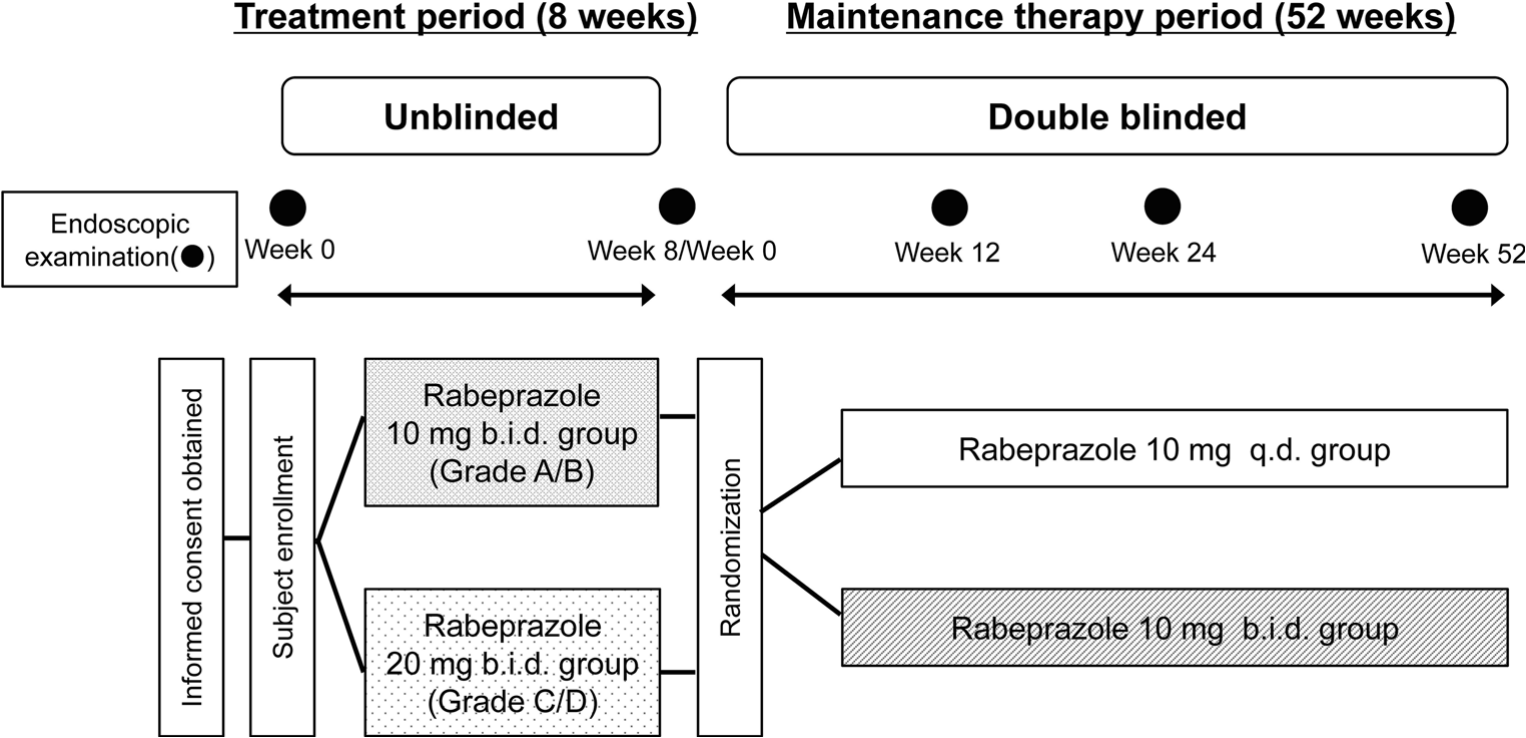
Study design.* q.d.* Once daily,* b.i.d.* twice daily

The study protocol, informed consent form, and their revisions were approved by the institutional review board and the director at each study site prior to study initiation. The study consisted of an 8-week treatment period followed by a 52-week maintenance therapy period. During the treatment period, patients with PPI-refractory RE, that is refractory to the standard once-daily PPI regimen, were treated with rabeprazole 10 or 20 mg b.i.d. in an open-label design. During the maintenance therapy period, subjects with endoscopically confirmed healing during the treatment period were randomized to receive rabeprazole 10 mg q.d. or 10 mg b.i.d. (Fig. 1).

The dose level during the treatment period was assigned according to the disease grading of the Los Angeles Classification [21, 22] based on endoscopic findings (physician’s assessment) at treatment entry: 10 mg b.i.d. for subjects with Grade A or B and 20 mg b.i.d. for those with Grade C or D. Subject allocation during maintenance therapy was determined by a third-party organization (Bell Medical Solutions, Tokyo, Japan), using the endoscopic findings at treatment entry as factors for stratified allocation; the subjects were evenly randomized to the 10 mg q.d. and 10 mg b.i.d. groups. During the maintenance therapy period, we used placebo tablets which had exactly the same appearance as the rabeprazole 10 mg tablets, the active drug. Subjects in the q.d. group took the active drug in the morning and placebo tablets in the evening, while subjects in the b.i.d group took the active drug both in the morning and in the evening. Subjects, investigators, and all other clinical study staff members were blinded to patient assignment during the maintenance therapy period.

Upper gastrointestinal endoscopy was performed using the Los Angeles Classification for eligibility assessment at treatment entry, evaluation of healing at Week 8 of treatment (before entering maintenance therapy), and evaluation of recurrence at Weeks 12, 24, and 52 of maintenance therapy. Subjects endoscopically confirmed to have unhealed disease at Week 8 of treatment were withdrawn, and those endoscopically confirmed to have recurrence during maintenance therapy completed the study upon confirmation.

Concomitant use of drugs possibly affecting the efficacy assessment, as well as contraindicated drugs, including PPIs, potassium-competitive acid blockers, H_2_ receptor antagonists, gastrointestinal prokinetic agents, protease inhibitors, sodium alginate, atazanavir sulfate, and rilpivirine hydrochloride, were prohibited throughout the trial.

This trial was initiated in September 2013 and completed in May 2016.

### Subjects

We enrolled patients with PPI-refractory RE, endoscopically confirmed to show no healing after at least 8 weeks of treatment with PPI at a standard q.d. dosing regimen approved in Japan, or even during maintenance therapy. Specifically, the standard doses were set at 10 or 20 mg/day for rabeprazole (double dose was allowed), 30 mg/day for lansoprazole, 20 mg/day for omeprazole, and 20 mg/day for esomeprazole. At the time of performing endoscopic examination for study enrollment, patients were examined for hiatal hernia, according to the diagnostic criteria proposed by Makuuchi et al. [23], as well as for gastric polyps.

Patients were excluded if they had any of the following conditions: upper gastrointestinal tract bleeding within 8 weeks prior to study enrollment (including ongoing bleeding at enrollment); any serious disease, such as Barrett’s esophagus (≥ 3 cm), Zollinger–Ellison syndrome, active gastric/duodenal ulcer; prior *H. pylori* eradication therapy within 6 months; existing or history of allergy to PPI; history of esophageal surgery or any other surgical intervention possibly affecting gastric acid secretion.

### Endpoints

The primary endpoint was the no-recurrence rate based on endoscopic findings at Week 52 of maintenance therapy, as assessed by the Central Assessment Committee consisting of three endoscopists (MK, MK, and MF). These endoscopists performed their central assessments independently and then discussed the results if there were differences among them in the evaluation results.

The secondary endpoints were the no-recurrence rate based on physician-assessed endoscopic findings, the period from randomization to recurrence, and the time-course changes in the incidence and the resolution rate of heartburn (daytime, nighttime). The presence or absence of heartburn was assessed by the investigators during medical interviews. The heartburn incidence during each of the 7-day periods immediately before visiting the hospital was assessed on a scale of five stages based on the number of days with symptoms: 0 (no symptoms), 1–2, 3–4, 5–6, and 7 (all) days. The incidence was tabulated by an analysis classifying the stages into two groups: “no symptom group” (0 days with symptoms) and “with symptoms group” (1 day or more with symptoms).

### Statistical analysis

Assuming no-recurrence rates of 80 and 60% in the rabeprazole 10 mg b.i.d. and q.d. dosing groups, respectively, a sample size of 218 patients (109 per group) was required to detect superiority of the b.i.d. regimen with a power of 90% and a two-sided significance level of 5% using the χ^2^ test. Thus, the target sample size was set at 300 for maintenance therapy, taking into consideration an approximately 25% withdrawal rate due to adverse events and other issues during the 52-week maintenance therapy period.

When determining the target sample size for the treatment, the healing rate of 75% was assumed to be appropriate for this period, based on the TWICE Study results [16]. Accordingly, a target sample size of 400 was set for the treatment to secure 300 patients for maintenance therapy. The protocol allowed enrollment of additional patients during the treatment period, if the number of patients eligible for enrollment in maintenance therapy failed to reach 300.

Analysis was performed on the primary endpoint, the no-recurrence rate based on endoscopic findings at Week 52 of maintenance therapy, using the χ^2^ test, to demonstrate superiority of the 10 mg b.i.d. regimen to the 10 mg q.d. regimen with a two-sided significance level of 5%. The analysis set included patients who completed maintenance therapy and were endoscopically confirmed to have recurrence during maintenance therapy and thereby withdrew from the study before Week 52. Furthermore, for the sensitivity analysis of the no-recurrence rate based on endoscopic findings at Week 52 of maintenance therapy, the Cochran Mantel–Haenszel (CMH) test was performed, using the disease grading according to the Los Angeles Classification (Grade A, B, C, or D) as a stratification factor, based on the endoscopic findings at treatment entry.

Similar analyses were performed on the physician’s assessment, inter-group comparisons, and heartburn incidence. Furthermore, Kaplan–Meier curves were generated by plotting data from randomization to recurrence.

## Results

### Characteristics and demographics of subjects

**Fig. 2:**
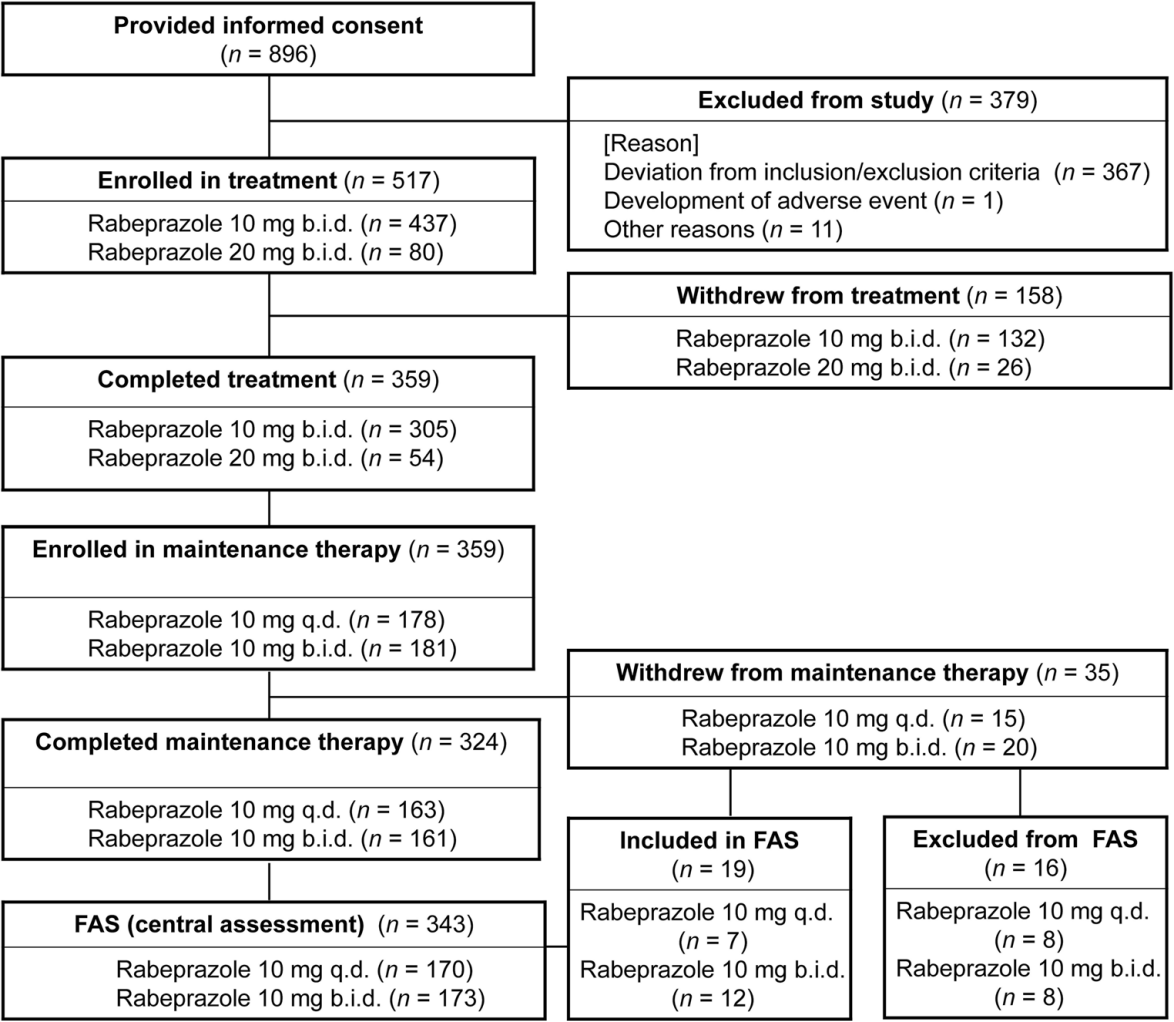
Patient disposition based on central assessment. *FAS* Full analysis set

Of the 896 patients giving consent, 517 met the eligibility criteria and began treatment, of whom 359 (69.4%) completed the treatment (Fig. 2). The remaining 158 subjects (30.6%) withdrew from treatment. The primary reason for withdrawal was a deviation from the inclusion criteria for the treatment (78 subjects) or being endoscopically confirmed as not having achieved healing at Week 8 of treatment (52 subjects). In a later review by the Central Assessment Committee, 78 subjects who had been considered ineligible for inclusion were judged to have Barrett’s esophagus or (RE-related) mucosal break that had already been cured.

In total, 359 subjects began maintenance therapy, of whom 324 (90.3%) completed the study. The remaining 35 subjects (9.7%) withdrew from maintenance therapy, but 19 were included in the central assessment full analysis set (FAS). The primary reasons for withdrawal were adverse events (10 subjects), personal issues (9 subjects), and other (not having achieved healing at Week 8 of treatment in the central assessment, 9 subjects; discrepancy in evaluations between physicians and the Central Assessment Committee, 4 subjects; need for prohibited concomitant therapy, 1 subject; non-compliance with protocol, 1 subject; pregnancy, 1 subject). Thus, 343 subjects constituted the central assessment FAS, the demographics of which are summarized in Table 1, which shows that there were no significant differences between the q.d. and b.i.d. dosing groups.

**Table 1:** Demographics and baseline characteristics of subjects during the maintenance therapy period (central assessment full analysis set)

Parameter	10 mg q.d. (*n* = 170)	10 mg b.i.d. (*n* = 173)	Total (*n* = 343)	*p* value^a^
Age (years)^b^				0.6612
< 65	71 (41.8)	68 (39.3)	139 (40.5)	
≥ 65	99 (58.2)	105 (60.7)	204 (59.5)	
Sex				0.3748
Male	102 (60.0)	112 (64.7)	214 (62.4)	
Female	68 (40.0)	61 (35.3)	129 (37.6)	
Body mass index (kg/m^2^)				0.6630
< 25.0	99 (58.2)	96 (55.5)	195 (56.9)	
≥ 25.0	71 (41.8)	77 (44.5)	148 (43.1)	
History of RE prior to treatment entry				0.3005
No healing after ≥ 8 weeks of PPI q.d.	147 (86.5)	142 (82.1)	289 (84.3)	
Recurred during PPI maintenance therapy	23 (13.5)	31 (17.9)	54 (15.7)	
Los Angeles Classification of reflux esophagitis at treatment entry				0.7765
Grade A	84 (49.4)	81 (46.8)	165 (48.1)	
Grade B	57 (33.5)	56 (32.4)	113 (32.9)	
Grade C	27 (15.9)	32 (18.5)	59 (17.2)	
Grade D	2 (1.2)	4 (2.3)	6 (1.7)	
Spinal deformity				0.3714
Yes	23 (13.5)	30 (17.3)	53 (15.5)	
No	147 (86.5)	143 (82.7)	290 (84.5)	
Esophageal hiatal hernia classification				0.2817
O (absent)	37 (21.8)	29 (16.8)	66 (19.2)	
C	28 (16.5)	22 (12.7)	50 (14.6)	
B	76 (44.7)	95 (54.9)	171 (49.9)	
A	29 (17.1)	27 (15.6)	56 (16.3)	
Heartburn at maintenance therapy entry				
Daytime				1.0000
Yes	20 (11.8)	21 (12.1)	41 (12.0)	
No	150 (88.2)	152 (87.9)	302 (88.0)	
Nighttime				0.1920
Yes	14 (8.2)	8 (4.6)	22 (6.4)	
No	156 (91.8)	165 (95.4)	321 (93.6)	
Daytime or nighttime				0.7607
Yes	26 (15.3)	24 (13.9)	50 (14.6)	
No	144 (84.7)	149 (86.1)	293 (85.4)	
Sleep disorder due to nocturnal heartburn or acid regurgitation at maintenance therapy entry				0.4995
Yes	5 (2.9)	3 (1.7)	8 (2.3)	
No	165 (97.1)	170 (98.3)	335 (97.7)	
Serum gastrin level at maintenance therapy entry (pg/mL)				0.2036
< 200	51 (30.0)	39 (22.5)	90 (26.2)	
≥ 200 to < 400	58 (34.1)	58 (33.5)	116 (33.8)	
≥ 400	61 (35.9)	76 (43.9)	137 (39.9)	
CYP2C19 genotype^c^				0.2941
Homozygous EM	78 (45.9)	72 (41.6)	150 (43.7)	
Heterozygous EM	79 (46.5)	79 (45.7)	158 (46.1)	
PM	13 (7.6)	22 (12.7)	35 (10.2)	
Anti-*Helicobacter pylori* immunoglobulin G antibody^d^				0.3685
Positive	8 (4.7)	13 (7.5)	21 (6.1)	
Negative	162 (95.3)	160 (92.5)	322 (93.9)	

Categorical variable data are presented as numbers of subjects. Values in parentheses are the proportion (%) of subjects in each dosing group relative to the number of subjects in the analysis set. The calculation excluded subjects with missing data

*PPI* proton pump inhibitor, q.d. once daily, *CYP* cytochrome P450, *EM* extensive metabolizer,* PM* poor metabolizer

^a^Fisher’s exact test was used

^b^Age at consent acquisition

^c^CYP2C19 genotype: *1/*1 was homozygous EM, *1/*2 and *1/*3 were heterozygous EM, *2/*2, *3/*3, and *2/*3 were PM

^d^Titer: ≥ 10 U/mL was defined as positive, and < 10 U/mL, as negative

### Efficacy

The healing rate based on endoscopic findings at Week 8 was 87.4% (362/414 subjects) during the treatment period, for the central assessment FAS. The healing rate stratified by treatment regimen was 88.9% (304/342 subjects) for the 10 mg b.i.d. and 80.6% (58/72 subjects) for the 20 mg b.i.d. regimen.

**Fig. 3:**
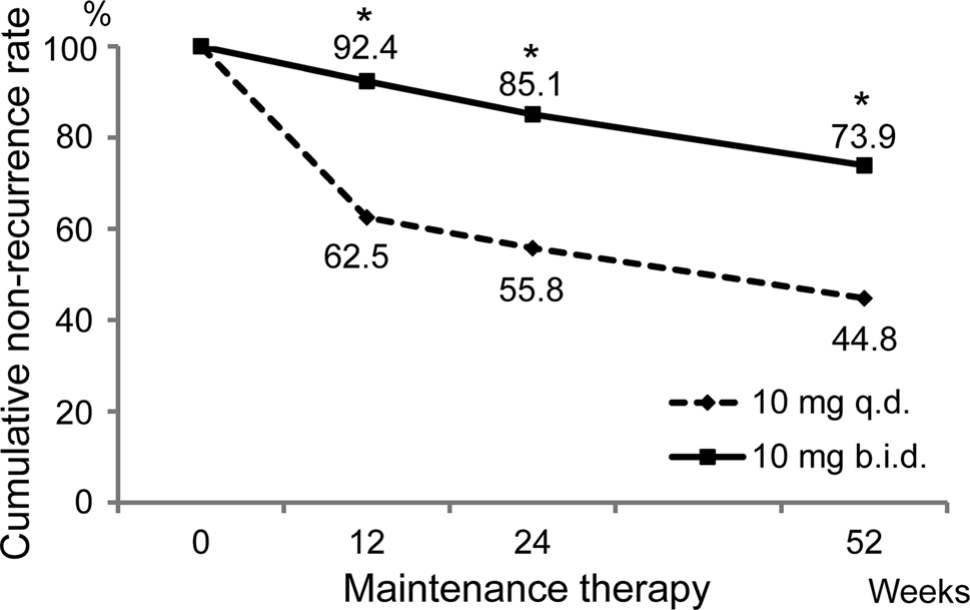
Primary endpoint: no-recurrence rate based on endoscopic findings at Week 52 of maintenance therapy per central assessment. The no-recurrence rate was analyzed using the χ^2^ test, and the superiority of the rabeprazole 10 mg b.i.d. regimen to the 10 mg q.d. regimen was verified

The no-recurrence rate based on endoscopic findings at Week 52 of maintenance therapy, the primary endpoint, was 44.8% (73/163 subjects) in the 10 mg q.d. group and 73.9% (119/161 subjects) in the 10 mg b.i.d. group in the central assessment FAS; the no-recurrence rate was significantly higher in the 10 mg b.i.d. group than in the 10 mg q.d. group (*p* < 0.001, χ^2^ test). This significant difference emerged at Week 12 of maintenance therapy (Fig. 3).

The sensitivity analysis using the CMH test showed significantly higher values in the 10 mg b.i.d. group than in the 10 mg q.d. group, results which were consistent with those observed for the primary endpoint.

The no-recurrence rate based on physicians’ assessments was significantly higher in the 10 mg b.i.d. group (75.9%, 120/158 subjects) than in the 10 mg q.d. group (49.7%, 77/155 subjects) (*p* < 0.001, χ^2^ test). These rates were in line with the central assessment results.

The cumulative no-recurrence rate at Week 52 estimated by the Kaplan–Meier method was significantly higher in the 10 mg b.i.d. group (71.4%) than in the 10 mg q.d. group (41.5%) (*p* < 0.001, log-rank test; hazard ratio, 0.34).

**Fig. 4:**
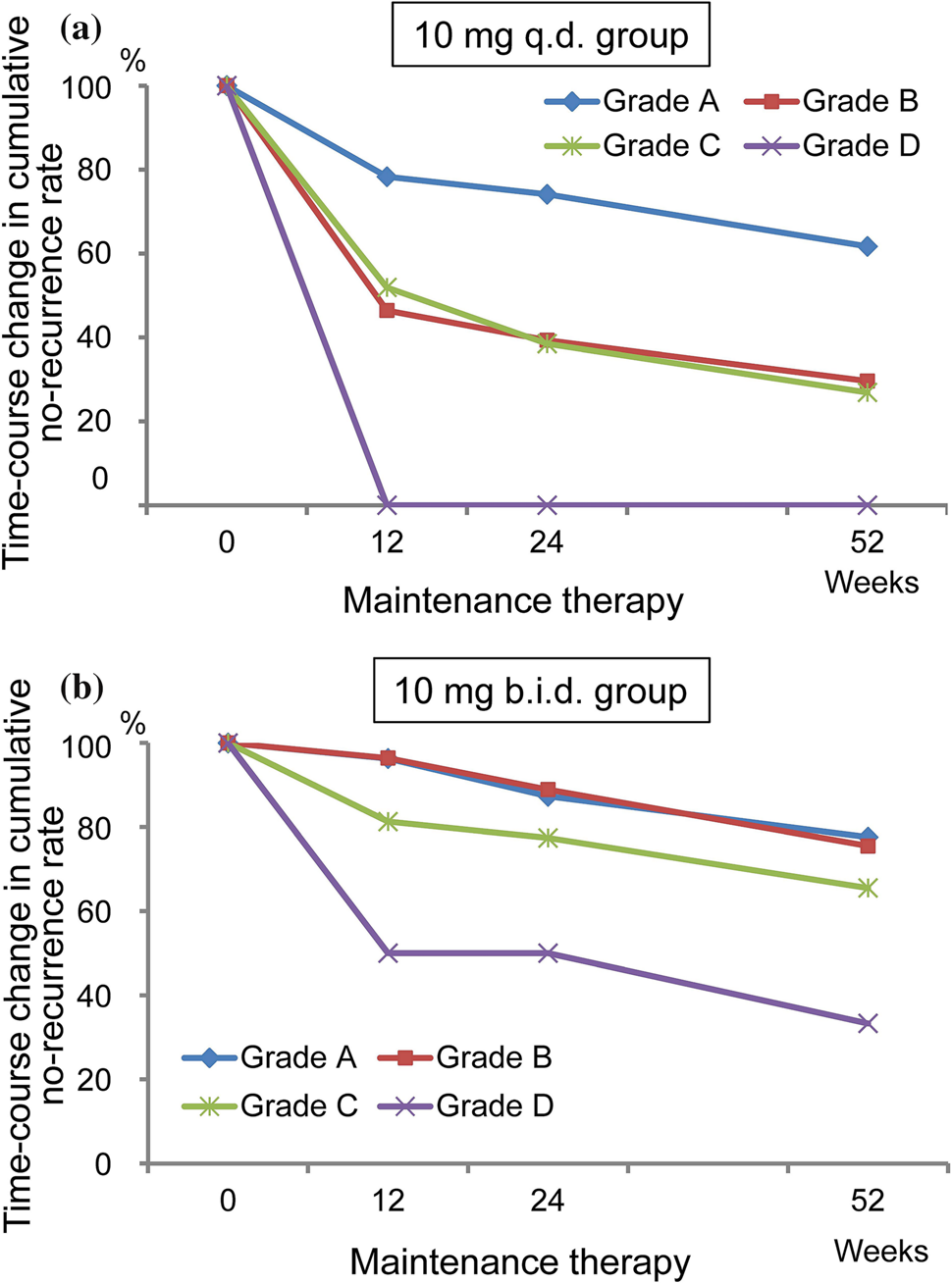
Stratified analysis: time-course change in no-recurrence rate at Week 52 of maintenance therapy stratified by the Los Angeles Classification at treatment entry. **a** 10 mg q.d. group, **b** 10 mg b.i.d. group

In the subgroup analyses of the primary endpoint stratified by subject background characteristics, the time-course change by RE severity (Los Angeles Classification) at treatment entry revealed a higher preventive effect in the b.i.d. group (no-recurrence rate ≥ 75% in subjects with Grade A/B, ≥ 65% in those with Grade C) than in the q.d. group (Fig. 4).

**Fig. 5:**
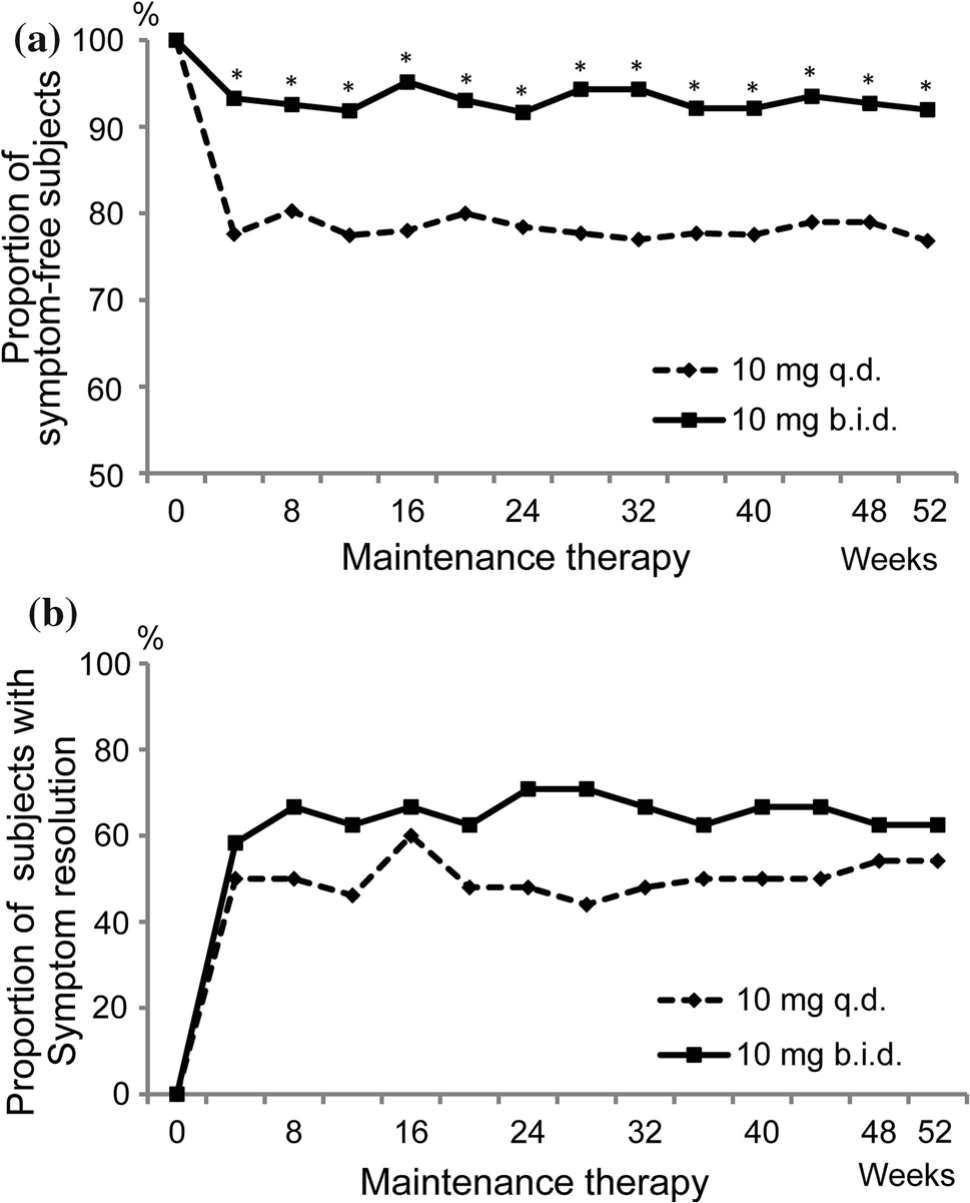
Secondary endpoint: time-course changes in suppression and occurrence rates of symptoms. **a** Proportion of symptom-free subjects (daytime/nighttime combined) among those without heartburn at maintenance therapy entry. **b** Proportion of subjects experiencing symptom resolution (daytime/nighttime combined) among those with heartburn at maintenance therapy entry

Analyses of heartburn incidences during the daytime and the nighttime were conducted in subjects free of symptoms at maintenance therapy entry. The proportion of symptom-free subjects was maintained at the same level throughout the day (daytime-nighttime combined) and was significantly higher in the 10 mg b.i.d. group than in the 10 mg q.d. group, with symptom-free rates at Week 52 of 92.0% (126/137 subjects) and 76.8% (106/138 subjects) (*p* < 0.001, χ^2^ test) (Fig. 5a). Analyses of the resolution rate of heartburn during the daytime and the nighttime were conducted in subjects who had symptoms at maintenance therapy entry. The resolution rate of heartburn throughout the day tended to be higher in the 10 mg b.i.d. group (62.5%, 15/24 subjects) than in the 10 mg q.d. group (54.2%, 13/24 subjects) (Fig. 5b).

### Safety

The incidence of adverse drug reactions was 3.9% (7/178 subjects) for the 10 mg q.d. group and 6.1% (11/181 subjects) for 10 mg b.i.d. group. Adverse drug reactions with an incidence of at least 1% included stomatitis in the 10 mg q.d. group, and diarrhea, elevated blood pressure, and increased blood thyroid-stimulating hormone in the 10 mg b.i.d. group.

Serum gastrin levels (mean ± standard deviation; upper limit of normal range 200 pg/mL) at treatment entry were 297.4 ± 302.16 and 321.8 ± 284.91 pg/mL in the 10 mg b.i.d and 20 mg b.i.d. groups, respectively. Serum gastrin levels at maintenance therapy entry were 359.1 ± 256.63 and 404.1 ± 304.96 pg/mL, and those at Week 52 of maintenance therapy were 283.3 ± 228.23 and 432.8 ± 253.83 pg/mL, in the 10 mg q.d. and 10 mg b.i.d. groups, respectively.

The cumulative gastric polyp incidence at Week 52 of maintenance therapy was 6.4% for the 10 mg q.d. group and 13.3% for the 10 mg b.i.d. group (Table 2). Gastric polyp types that developed by Week 52 in the 10 mg q.d. group and the 10 mg b.i.d. group included fundic gland polyps (5.8 and 8.7%, respectively), gastric hyperplastic polyps (1.2 and 4.0%) and other types (0 and 1.2%) (note: some subjects were included in multiple categories).

**Table 2:** Cumulative gastric polyp incidence at Week 52 of maintenance therapy (safety analysis set)

Category	Therapy		*p* value^a^
	10 mg q.d. (*n* = 178)	10 mg b.i.d. (*n* = 181)	
Gastric polyp present^b^ (%)	11 (6.4)	23 (13.3)	0.0457
New gastric polyp developed	11 (6.4)	19 (11.0)	0.1805
Existing gastric polyp increased/enlarged	1 (0.6)	7 (4.0)	0.0672
Gastric polyp present during the treatment period^b^	1 (0.6)	9 (5.2)	–
New gastric polyp developed	1 (0.6)	5 (2.9)	–
Existing gastric polyp increased/enlarged	0 (0.0)	6 (3.5)	–
Gastric polyp absent at maintenance therapy entry^b^	10 (5.8)	14 (8.1)	–
New gastric polyp developed	10 (5.8)	14 (8.1)	–
Existing gastric polyp increased/enlarged	1 (0.6)	1 (0.6)	–
Gastric polyp absent (%)	160 (93.6)	150 (86.7)	–

Subjects with gastric polyp data available for assessment in the safety analysis set were included in the analysis. Values are presented as the number of subjects, with the proportion (%) of subjects in each dosing group given in parenthesis

^a^Fisher’s exact test was used

^b^Some subjects were included in multiple gastric polyp categories

## Discussion

In the treatment of RE, prevention of acid regurgitation into the esophagus, healing of mucosal breaks, and maintenance of mucosal healing status constitute the most important strategy for resolving subjective symptoms, improving QOL [24], and preventing complications [25]. Severe RE that is refractory to a standard PPI regimen is associated with more frequent nocturnal reflux symptoms and sleep disorder due to longer retention of regurgitated acid in the esophagus [26]. Progression into more severe esophageal acid reflux is associated with an increased incidence of Barrett’s esophagus, advancement to esophageal adenocarcinoma, and the complications of esophageal hemorrhage and stenosis [27, 28]. Because PPI therapy is a form of supportive care for acid regurgitation, PPI-refractory RE with severe acid regurgitation requires potent inhibition of acid secretion by means of ongoing maintenance therapy.

This study registered patients with PPI-refractory RE who were assessed by physicians as not having achieved a cure after at least 8 weeks of treatment with a standard PPI regimen. However, the Central Assessment Committee later reviewed the subjects’ eligibilities, and disqualified 78 judged to have Barrett’s esophagus, or RE lesions that had already been cured, thus making them ineligible for this study. In the protocol of this study, short-distance, whole circumference endoscopic images of the gastroesophageal junction were to be submitted to the Central Assessment Committee, and an average of 3.3 endoscopic images per patient were submitted to the Central Assessment Committee during each assessment period. If there were differences in the evaluation results among the three endoscopists in the Central Assessment Committee, a final agreement was reached after discussions among the three endoscopists.

The no-recurrence rate, based on endoscopic findings at Week 52 of maintenance therapy per central endoscopy assessment, was significantly higher in the 10 mg b.i.d. group than in the 10 mg q.d. group. The CMH test, which was carried out from the sensitivity analysis viewpoint with adjustment by the Los Angeles Classification based on endoscopic findings at treatment entry, revealed no difference from the primary results, i.e. the 10 mg b.i.d. group showed significant superiority to the 10 mg q.d. group. A previous study conducted by Shimatani et al. in *H. pylori*-negative healthy adults demonstrated intragastric pH > 4 holding time ratios of 49, 59 and 71% in homo EM, hetero EM, and poor metabolizer (PM) subgroups, respectively, with a rabeprazole 10 mg q.d. regimen, as well as ratios of 85, 86, and 99%, respectively, with a rabeprazole 10 mg b.i.d. regimen [29]. In the current trial, the inhibitory effects on acid secretion also correlated with the mucosal healing effect during maintenance therapy. Similar tendencies were indicated in the current trial between the central assessment and physicians’ assessments in the endoscopic recurrence evaluation during maintenance therapy. Although evaluator-associated variability was reported for endoscopic diagnosis [30], such variation was limited in the current trial which employed sample image collection to achieve consistent assessment. In the protocol of this study, we had stipulated that the dosing timing of rabeprazole was to be “after breakfast” and “after dinner”. Thus, we are confident that we were able to compare the q.d. and b.i.d. regimens in an appropriate manner. It has also been shown that acid secretion inhibition by rabeprazole is not affected by meals [31]. We therefore believe that a proper treatment effect was exerted.

In our subgroup analyses, stratification by RE severity at treatment entry revealed a recurrence rate exceeding 70% in Grades B to D subjects in the 10 mg q.d. group. These observations support recommending a 10 mg b.i.d. regimen as maintenance therapy for patients with PPI-refractory RE of Grade B or higher. Similarly, the no-recurrence rate was higher in the 10 mg b.i.d. group than in the 10 mg q.d. group of patients with PPI-refractory RE with severe esophageal hiatal hernia which is known to be a cause of PPI-refractory RE [32].

In the evaluation of subjective symptoms, the proportion of symptom-free subjects (daytime-nighttime combined) was also higher in the b.i.d. than in the q.d. group, showing the superiority of the b.i.d. regimen to the q.d. regimen for the prevention of symptom onset. The symptom-free rate for heartburn changed until Week 8 of the maintenance therapy period, which might have been attributable to dose reduction from the treatment period to the maintenance therapy period. However, after Week 8, the symptom-free rate remained essentially stable until the end of the maintenance therapy period. In particular, we observed that the heartburn symptoms appeared to be more likely to have developed in the groups in which the dose was changed from 20 mg b.i.d.to 10 mg b.i.d., from 20 mg b.i.d. to 10 mg q.d., or from 10 mg b.i.d. to 10 mg q.d.

The incidences of adverse drug reactions did not differ markedly between the two groups during the maintenance therapy period. Furthermore, long-term treatment with the b.i.d. regimen did not increase incidences of any specific events or the development of new events: the incidences were lower than those in a previous study evaluating rabeprazole 10 mg or 20 mg q.d. maintenance therapy for RE patients [8].

The target population of this study was PPI-refractory RE patients previously treated with PPIs for at least 8 weeks, and the subjects therefore had higher serum gastrin levels at the start of treatment. Following treatment initiation, serum gastrin levels increased slightly, but the mean serum gastrin levels remained stable without further increase during the maintenance therapy period. These results are consistent with those of a previous study [16], and the values were lower than in patients treated with potassium-competitive acid blockers [33].

The cumulative gastric polyp incidence was higher in the 10 mg b.i.d. group (13.3%) than in the 10 mg q.d. group (6.4%). On the other hand, the cumulative gastric polyp incidence at Week 52 estimated by the Kaplan–Meier method in the 10 mg b.i.d. group was 15.7%, slightly higher than the 12.7% in the 10 mg q.d. group. These incidences were not markedly higher than those reported in a prospective study evaluating a 10 mg q.d. maintenance therapy regimen for RE patients [34].

This study has three limitations: first, we did not evaluate the rabeprazole 20 mg b.i.d. regimen as maintenance therapy. Because approximately 26% of subjects experienced recurrence even during 52-week maintenance therapy with the rabeprazole 10 mg b.i.d. regimen, subjects with severe mucosal breaks (Los Angeles Classification Grades C and D) after being given a standard PPI regimen, and then received treatment with the 20 mg b.i.d. regimen, might have required the same dosing regimen during the maintenance therapy period. Second, the incidences of adverse events in the q.d. group might have been underestimated: the protocol specified study withdrawal upon endoscopic recurrence, which might have resulted in a shorter mean exposure to the study drug in the q.d. group which had a higher recurrence rate than the b.i.d. group. Third, the study period was 52 weeks such that no data are available on efficacy and safety beyond 52 weeks. Future investigation is warranted to evaluate the results of longer treatment.

In summary, this study demonstrated maintenance therapy with rabeprazole 10 mg b.i.d. up to Week 52 to be significantly superior to rabeprazole 10 mg q.d. in preventing RE recurrence in patients with PPI-refractory RE.
